# Label-free multiphoton imaging to assess neoadjuvant therapy responses in breast carcinoma

**DOI:** 10.7150/ijbs.41579

**Published:** 2020-02-21

**Authors:** Lianhuang Li, Zhonghua Han, Lida Qiu, Deyong Kang, Zhenlin Zhan, Haohua Tu, Jianxin Chen

**Affiliations:** 1 Key Laboratory of OptoElectronic Science and Technology for Medicine of Ministry of Education, Fujian Provincial Key Laboratory for Photonics Technology, Fujian Normal University, Fuzhou 350007, P. R. China; 2 Department of Breast Surgery, Fujian Medical University Union Hospital, Fuzhou 350001, P. R. China; 3 College of Physics and Electronic Information Engineering, Minjiang University, Fuzhou 350108, P. R. China; 4 Department of Pathology, Fujian Medical University Union Hospital, Fuzhou 350001, P. R. China; 5 Beckman Institute for Advanced Science and Technology, University of Illinois at Urbana-Champaign, Urbana, IL 61801, USA

**Keywords:** breast carcinoma, neoadjuvant chemotherapy, treatment response, multiphoton imaging

## Abstract

Neoadjuvant chemotherapy has been used increasingly in patients with early-stage or locally advanced breast carcinoma, and has been recommended as a general approach in locally advanced-stage diseases. Assessing therapy response could offer prognostic information to help determine subsequent nursing plan; particularly it is essential to identify responders and non-responders for the sake of helping develop follow-up treatment strategies. However, at present, diagnostic accuracy of preoperative clinical examination are still not satisfactory. Here we presented an alternate approach to monitor tumor and stroma changes associated with neoadjuvant therapy responses in breast carcinoma, with a great potential for becoming a new diagnostic tool—multiphoton microscopy. Imaging results showed that multiphoton imaging techniques have the ability to label-freely visualize tumor response such as tumor necrosis, and stromal response including fibrosis, mucinous response, inflammatory response as well as vascular hyperplasia *in situ* at cellular and subcellular levels. Moreover, using automated image analysis and a set of scoring methods, we found significant differences in the area of cell nucleus and in the content of collagen fibers between the pre-treatment and post-treatment breast carcinoma tissues. In summary, this study was conducted to pathologically evaluate the response of breast carcinoma to preoperative chemotherapy as well as to assess the efficacy of multiphoton microscopy in detecting these pathological changes, and experimental results demonstrated that this microscope may be a promising tool for label-free, real-time assessment of treatment response without the use of any exogenous contrast agents.

## Introduction

The use of chemotherapy as a form of neoadjuvant treatment for breast cancer is increasing. The advantages include tumor size decrease which would improve the resectability rate and allow a greater number of patients to undergo breast-conserving operation 1-3. Alterations of tumor burden and microenvironment in the presurgical setting are also associated with longer disease-free or overall survival in breast cancer patients 4, 5. As a result, the influences of primary chemotherapy on tumor morphology and biological features should be studied more carefully. Nevertheless, the accurate evaluation of treatment response still remains a great challenge 6, 7, and many women would receive chemotherapy unnecessarily. Though mammography is a more sensitive tool for diagnosing breast carcinoma, previous research has shown its limited effectiveness in monitoring changes in tumor size after primary chemotherapy. For instance, it was found to overestimate the residual tumor size and ignore tumor downstaging 8. Besides, stromal fibrosis induced by chemotherapy which was said to happen in up to 67% of tumors could radiologically mimic the malignant tissues and may lead to clinical overestimation of the size of residual lesions too 9, 10. At present, pathologic examination of the excised tumor tissues is the gold standard for estimating therapeutic outcomes 11, but diagnosis results can only be obtained postoperatively, and there is no guidance or help for the preoperative treatment.

On the other hand, tumor regression after treatment would be related with an increased incidence of multifocality as it may be caused by a process of tumor segmentation, and thereby mammography or preoperative clinical examinations are inadequate for the choice of candidates for breast conservation due to the limitation of resolution 8, 12. This requires a higher resolution imaging technique which can *in situ* visualize suspicious lesions at cellular and subcellular levels. Many optical imaging techniques, such as multiphoton microscopy and confocal laser scanning microscope, are emerging as powerful tools for high spatial-resolution and non-invasive monitoring of biological tissues 13, 14. Comparing with the confocal microscopy, multiphoton microscopy (MPM) has many advantages including label-free imaging, deeper penetration, low photodamage and phototoxicity as well as intrinsic optical sectioning, and therefore has been used in various imaging studies 15-17. At present, it is the preferred technique for subcellular observation of thick tissues and live animals 18, 19. To date, two-photon autofluorescence (TPAF) and second harmonic generation (SHG) are the most widely used imaging modalities involved with MPM. TPAF imaging has been applied to label-freely visualize cellular structure and behavior, while SHG imaging has been applied to detect the collagen fibers in extracellular matrix 20, 21. It is thus clear that MPM has potential to improve the detection of treatment response. Here, we present the use of TPAF and SHG imaging as a non-invasive method to label-freely evaluate the neoadjuvant chemotherapy response of breast carcinoma and then estimate the likelihood that this approach will work. To our knowledge, this is the first study using multiphoton imaging to assess the efficacy of neoadjuvant therapy for breast cancer.

## Materials and Methods

### Sample Preparation

This research was carried out with the approval of the institutional review board at the Fujian Medical University Union Hospital, and before participating in the study, each patient signed an informed consent. Our study group consisted of 70 women with advanced primary breast carcinoma, and we acquired 30 pre-therapy as well as 40 post-therapy fresh tumor tissues. For these patients who underwent preoperative treatment, they all received neoadjuvant chemotherapy consisting of 4 cycles of epirubicin (90 mg/m²) and cyclophosphamide (600 mg/m²) every 3 weeks, following by 4 cycles of docetaxel (100 mg/m²) every 3 weeks. Trastuzumab was given to HER2-positive patients at 8 mg/kg loading dose following by 6 mg/kg maintenance dose during the docetaxel treatment. Therapeutic anti-tumor effects were assessed according to the response evaluation criteria in solid tumors. Surgery was performed approximately 2 weeks after the last chemotherapy cycle. The decision for mastectomy or breast conserving surgery was based on the clinical data and patient's choice. All HER2-positive patients received concomitant intravenous trastuzumab 6 mg/kg every 3 weeks to complete 1-year treatment. Radiotherapy and hormone therapy were given in accordance with guidelines. For every specimen, two consecutive slices were obtained via a cryostat microtome (Thermo Scientific CryoStar NX50, USA), where one section (10 μm thickness) was used for imaging study, and an adjacent slide was stained with hematoxylin and eosin (H&E) for contrastive analysis. In the course of the experiment, we dripped small amounts of phosphate-buffered saline (PBS) on the slices in order to prevent dehydration or shrinkage. A bright field light microscope (Eclipse Ci-L, Nikon Instruments Inc., Japan) with a CCD (DS-Fi2, Nikon) was used to take the digital images of the H&E-stained sections. For the sake of confirmation, each image obtained from MPM was compared with the corresponding H&E-stained image which has been checked by a certified pathologist.

### Imaging Equipment

The multiphoton imaging device used in this research has been illustrated in our previous publications 22. In summary, a commercial microscope (LSM 880, Zeiss, Germany) which was combined with a mode-locked femtosecond Ti: sapphire laser (Chameleon Ultra, Coherent, Inc., USA) was applied to gain high-resolution multiphoton images. The 810 nm wavelength was selected as the excitation light and a 63×/1.4 Plan-Apochromat oil immersion lens was chosen in the experiment, and in this case, a lateral resolution of 0.3 micron as well as an axial resolution of 0.8 micron could be obtained, respectively. SHG signal in the wavelength range of 394-416 nm was collected via a GaAsP photomultiplier tube (PMT), while TPAF signal covered the wavelength range 430-759 nm was detected using a 32-channel GaAsP PMT array detector. The time to collect each pixel was 1.8 microsecond and thereby the scanning speed of this system was two 2 frames per second (512×512 pixels). Large-area MPM image was achieved by assembling an array of images with 512×512 pixels. To increase contrast, TPAF image was marked in red and SHG image was marked in green.

### Automatic Image Analysis

**Figure 1:**
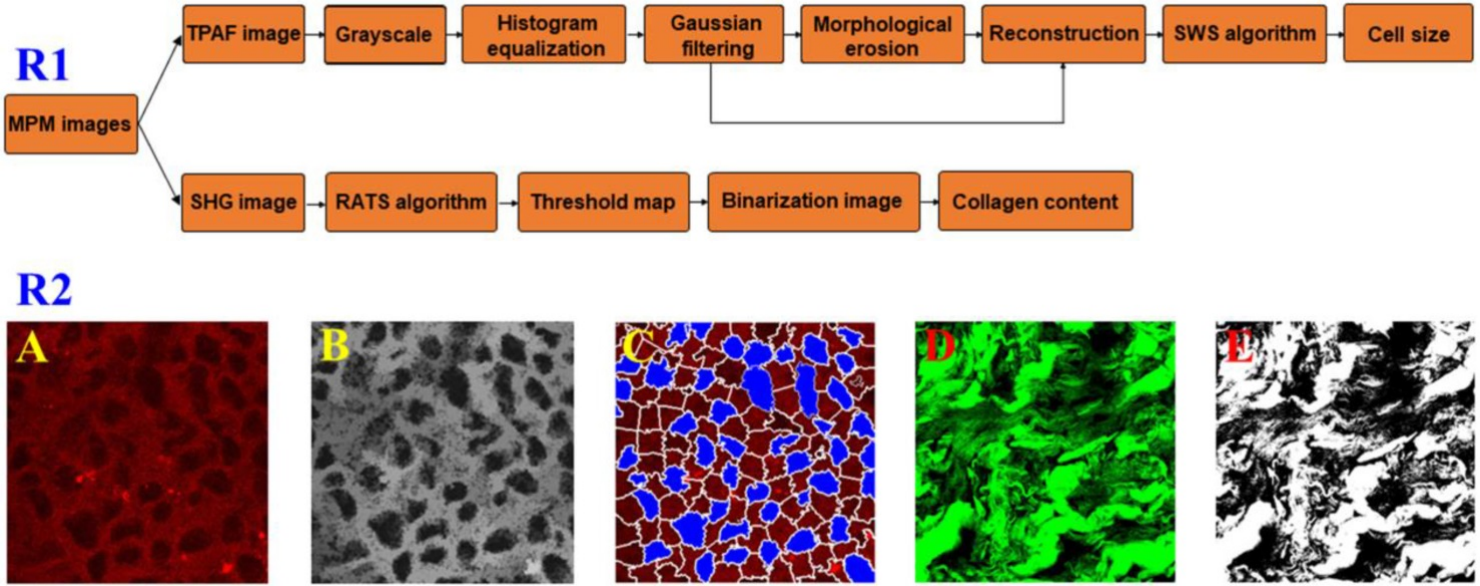
Row 1: a flowchart of automated image processing strategy for measuring cell size and collagen content; Row 2: a schematic of quantitative analysis from MPM images. (A) Original TPAF image; (B) Reconstruction image; (C) Segmentation result by SWS algorithm; (D) Original SHG image; and (E) Binarization image.

To test if there were changes in the cell size and collagen fibers between the pre- therapy and post- therapy tumors, we used automated image analysis to measure the area of cell nucleus and collagen content from MPM images and a detailed flowchart about the image processing strategies was present in Figure 1R1. In TPAF image, the cell nucleus was shown in black because it did not produce a signal and accordingly is easy to identify. As shown in Figure 1A-C, TPAF image was converted to grayscale and preprocessed with histogram equalization and Gaussian filtering, and then morphological erosion operation was performed to yield reconstructed image as the input of spatial-constrained watershed superpixel (SWS) algorithm, and finally, SWS indicated the location of cells by delineating their boundaries with corresponding superpixels and measured nuclear area by counting the number of pixels in the associated superpixel.

Additionally, we would achieve the collagen content from SHG image because only collagen fibers could produce SHG signal, and in this research work, the robust automatic threshold selection (RATS) algorithm was adopted for quantitatively calculating the collagen content. To be more specific, as shown in Figure 1D and E, SHG image was first converted to a grayscale image, then a corresponding threshold map which gave the optimal segmentation threshold for each image position associated with the collagen distribution region was calculated based upon the value of pixels and their gradient information, and collagen structure was segmented by binarizing grayscale image with thresholds, and finally in binarization image, the pixels with value 255 represented the collagen structure, and we can obtain the collagen content by computing the percentage of pixels with value 255 in the region of interest.

### Statistical Analysis

The IBM SPSS Statistics 21 was used for performing statistical analysis, and the student's t-test was used for assessing the statistical significance. *P-*value less than 0.05 was thought to be statistically significant.

## Results

### Multiphoton imaging of the pre-therapy tumors

**Figure 2:**
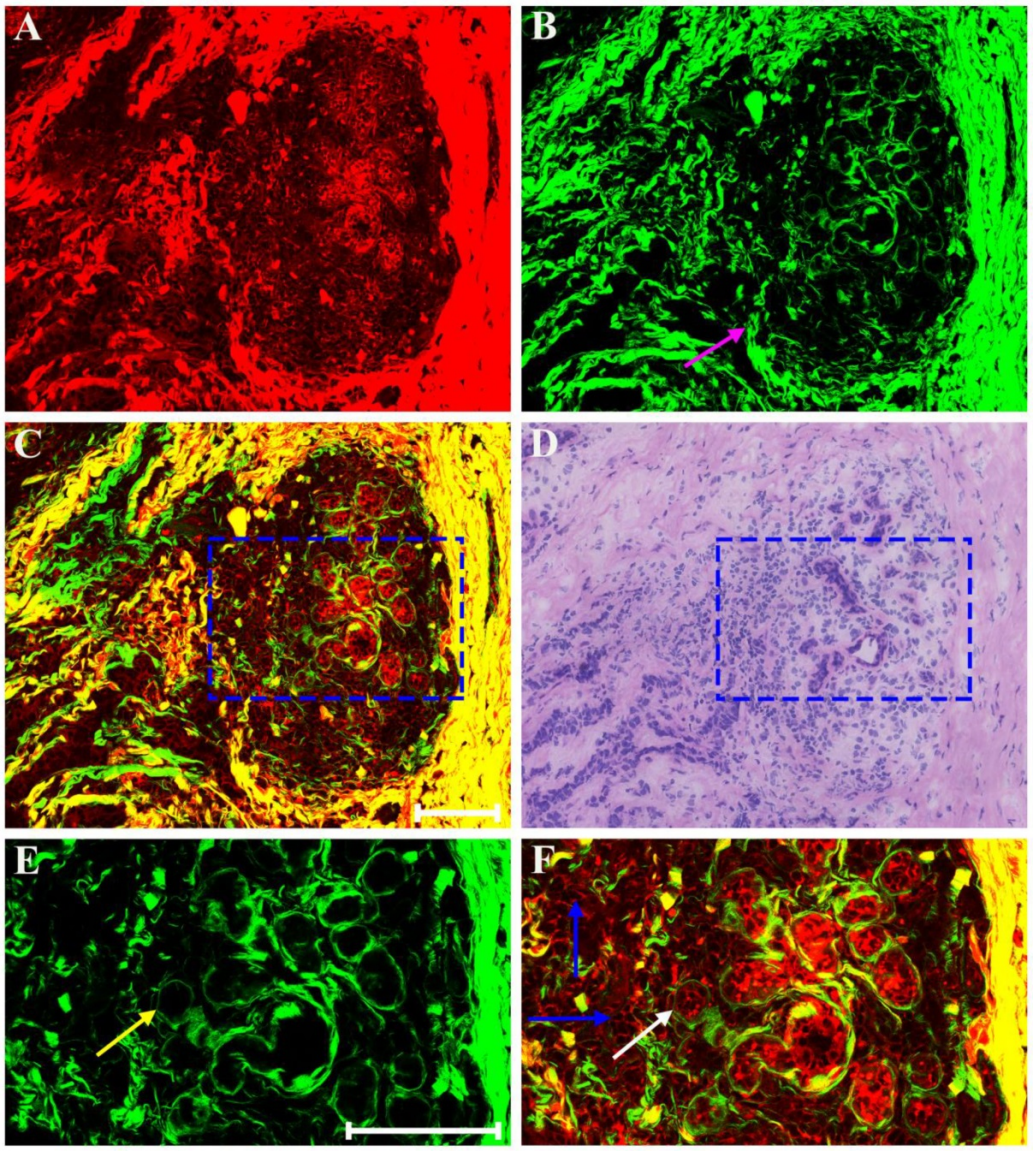
MPM images of breast lobule with tumor invasion and corresponding H&E-stained image. (A) TPAF image; (B) SHG image; (C) Overlaid image; (D) H&E-stained image; and (E-F) Zoom-in SHG and MPM images of the blue boxed region in (C) respectively. Pink arrow: broken collagen fibers; yellow arrow: basement membrane; white arrow: acinus; blue arrow: tumor cell. Scale bar: 100μm.

**Figure 3:**
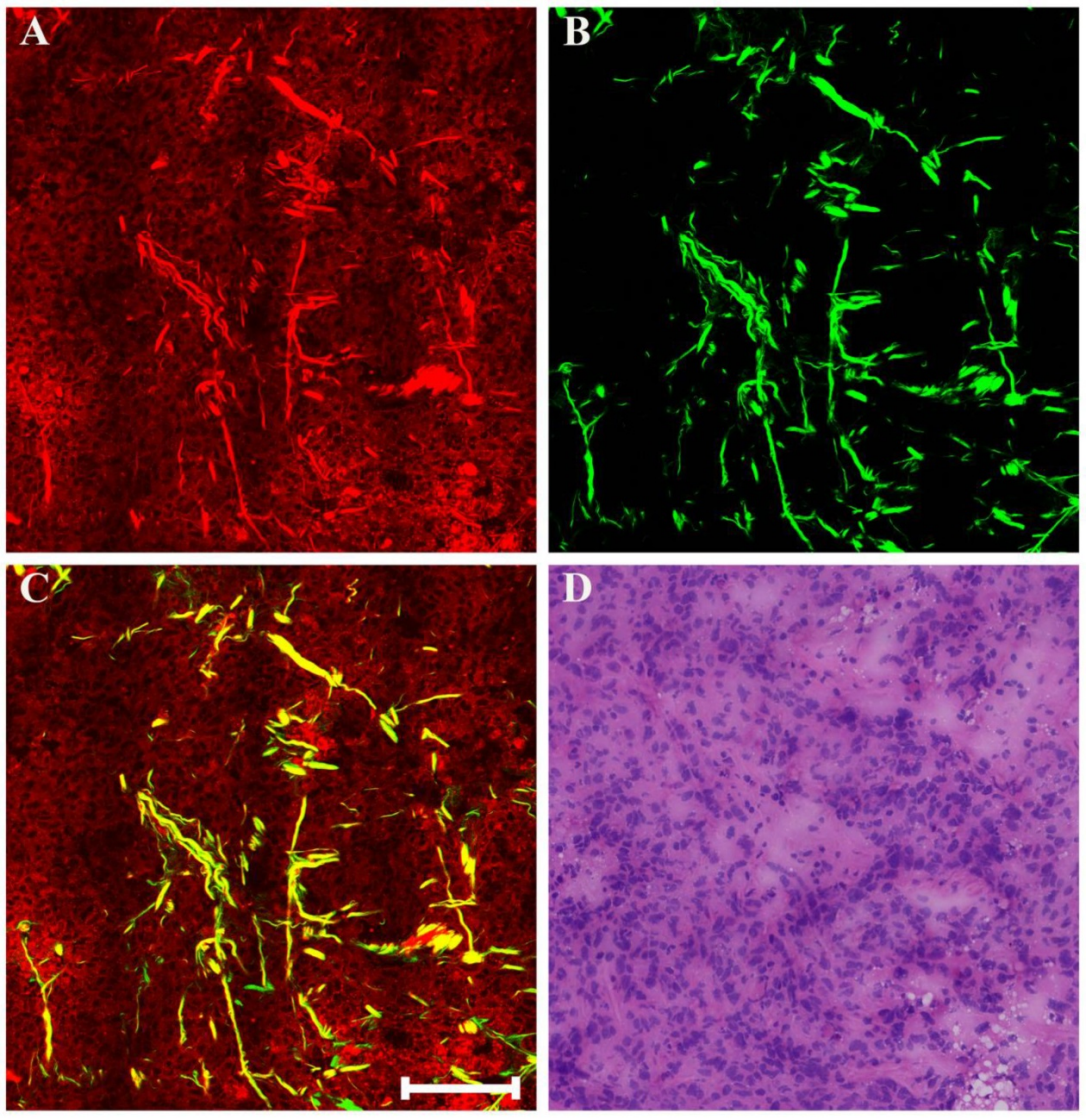
MPM images of the pre-treatment breast cancer and corresponding H&E-stained image. (A) TPAF image; (B) SHG image; (C) Overlaid image; (D) H&E-stained image. Scale bar: 100μm.

Clinical pathologists should have the ability to interpret morphologic alterations induced by preoperative treatment and differentiate them from tumor-intrinsic morphologic characteristics for correct histopathologic assessment. Thus, in this work, we first studied multiphoton imaging of breast tumor tissues for comparative analysis. Figure 2 shows representative MPM images of breast lobule with tumor invasion, and the digital image of the adjacent H&E-stained slice is given too for confirmation. TPAF image presents tumor cells invade into the lobular unit, and SHG image demonstrates collagen fibers surrounding the lobule are severely broken (pink arrow in Figure 2B), while overlaid image (Figure 2C) can show the spatial distribution of tissue microstructure more clearly. Single tumor cell (blue arrow in Figure 2F) can be detected by TPAF signal. In this region, normal ducts are absent, but breast lobule is still present, for example, acinus (white arrow in Figure 2F) can be clearly seen, and basement membrane surrounding the acinus (yellow arrow in Figure 2E) could be detected too via SHG signal. Thus, MPM imaging can clearly show the alterations in the microstructure of mammary tissues after tumor invasion. Moreover, MPM images of advanced breast cancer are displayed in Figure 3. The normal structure of mammary gland has been completely destroyed with the invasion of a large number of tumor cells. The collagen network from normal tissues disappears completely and only some residual broken collagen fibers can be found in the SHG image (Figure 3B). By comparing with the corresponding H&E-stained image (Figure 2D and Figure 3D), we could get a conclusion that on one hand, TPAF imaging is capable of real-time observation of cells and their subcellular components in intact tissues; on the other hand, SHG imaging is prominent to watch collagen fibers and is able to identify different patterns of collagen distribution in extracellular stroma which could provide complementary information about tissue microstructures and cannot be obtained directly with usual optical microscope of H&E-stained sections.

### Multiphoton imaging of the post-therapy tumors

**Figure 4:**
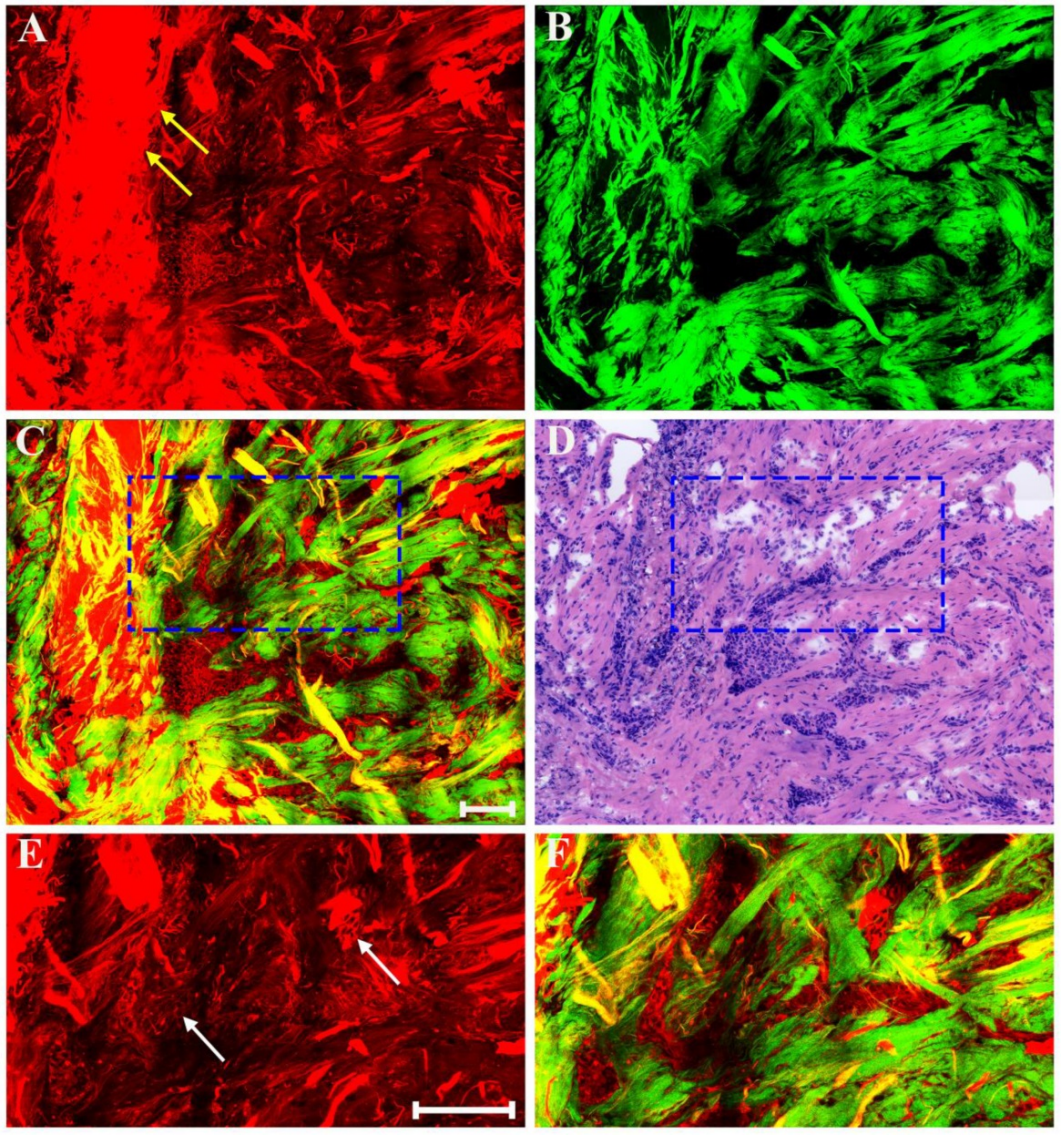
MPM images of the post-treatment breast cancer and corresponding H&E-stained image. (A) TPAF image; (B) SHG image; (C) Overlaid image; (D) H&E-stained image; and (E-F) Zoom-in TPAF and MPM images of the blue boxed region in (C) respectively. Yellow arrow: tumor necrosis; white arrow: residual tumor cells. Scale bar: 100μm.

Generally speaking, the morphologic changes induced by drug that may happen in the neoplastic tissues are of considerable importance, and the morphology of the tumor before and after chemotherapy should be compared. Therefore, our next work was to help identify these drug-caused histopathological changes using biomedical multiphoton microscopy. Following treatment tumor may show obvious regression, and thereby may present fibrous changes with little or no remaining carcinoma. The typical MPM images of breast carcinoma after neoadjuvant therapy are shown in Figure 4. Here the changes are seen in the residual tumor. TPAF image demonstrates that decreased nuclear density are present by comparing to the pre-treatment tumor tissues, and could show scanty pleomorphic carcinoma cells which are shown more clearly in the zoom-in image (white arrow in Figure 4E). These residual tumor cells tend to shrink away from their surrounding matrix. In addition, tumor necrosis (yellow arrow in Figure 4A) can be easily observed because it generates strong TPAF signal. SHG image presents a large number of tangled and bulky collagen fibers because of fibrosis which demonstrates marked response to neoadjuvant chemotherapy and would help confirm the original location of the malignant tumor. The overlaid image (Figure 4C) has obvious characteristics of rare tumor cells imperceptibly remaining in dense fibrotic stroma, and therefore would help to understand the spatial distribution of tissue components. Obviously, MPM can effectively monitor tumor response after treatment and accurately detect residual tumor cells.

**Figure 5:**
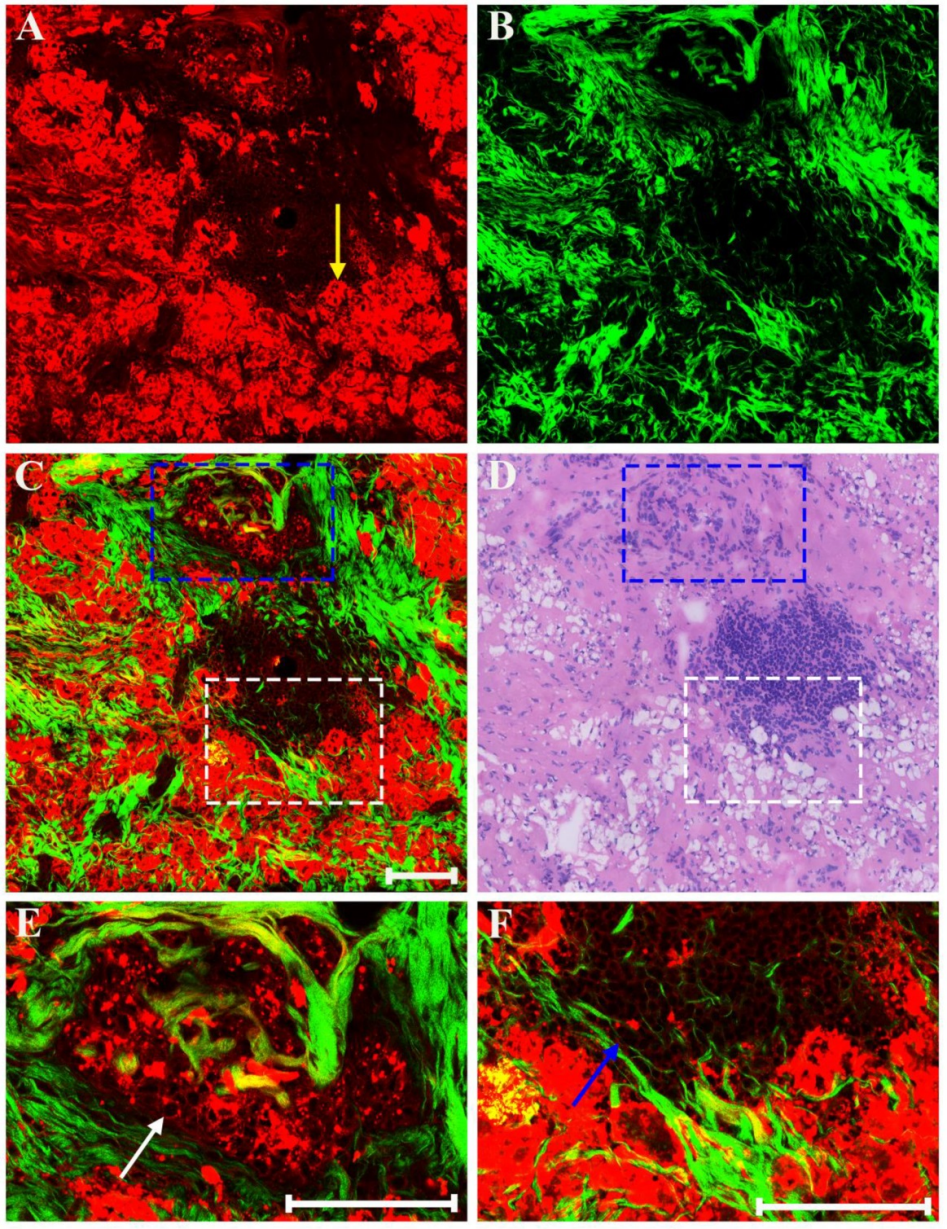
MPM images of inflammatory response in breast cancer after neoadjuvant treatment and corresponding H&E-stained image. (A) TPAF image; (B) SHG image; (C) Overlaid image; (D) H&E-stained image; and (E-F) Zoom-in MPM images of the blue and white boxed regions in (C) respectively. Yellow arrow: foamy cells; white arrow: small nest of malignant cells; blue arrow: lymphocytes. Scale bar: 100μm.

In addition, as a result of neoadjuvant treatment, there may be patchy inflammation within or around the fibrous matrix. Representative MPM images of inflammatory response in breast cancer after preoperative chemotherapy are presented in Figure 5. Morphologic changes in tumor tissues are obvious, such as, tumor cellularity is severely reduced, and only a small nest of malignant cells (white arrow in Figure 5E) is found in this area. Obviously, there is a marked inflammatory response in the stroma. Aside from fibrosis which is virtually universal, the stromal compartment shows lots of lymphocytes and foamy cells. The lymphocytes (blue arrow in Figure 5F) flock together, and are dark because these cells have less cytoplasm and therefore generate a relatively weak TPAF signal; by contrast, the foamy cells (yellow arrow in Figure 5A) is obvious because large amounts of lipids accumulate in the cytoplasm of these cells and thus they can emit strong TPAF signal.

**Figure 6:**
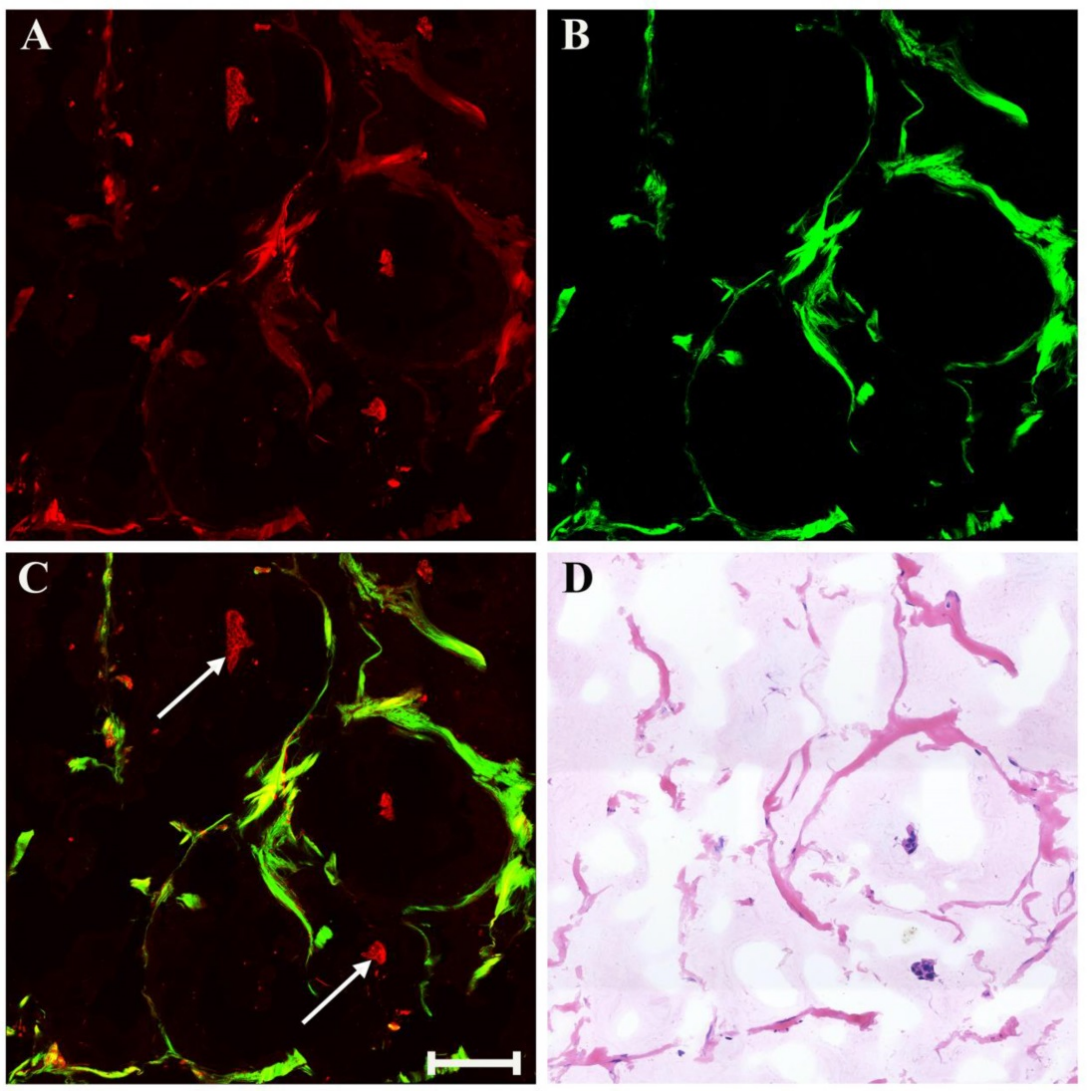
MPM images of mucinous response in breast cancer after neoadjuvant treatment and corresponding H&E-stained image. (A) TPAF image; (B) SHG image; (C) Overlaid image; (D) H&E-stained image. White arrow: residual tumor cells floating in mucus. Scale bar: 100μm.

**Figure 7:**
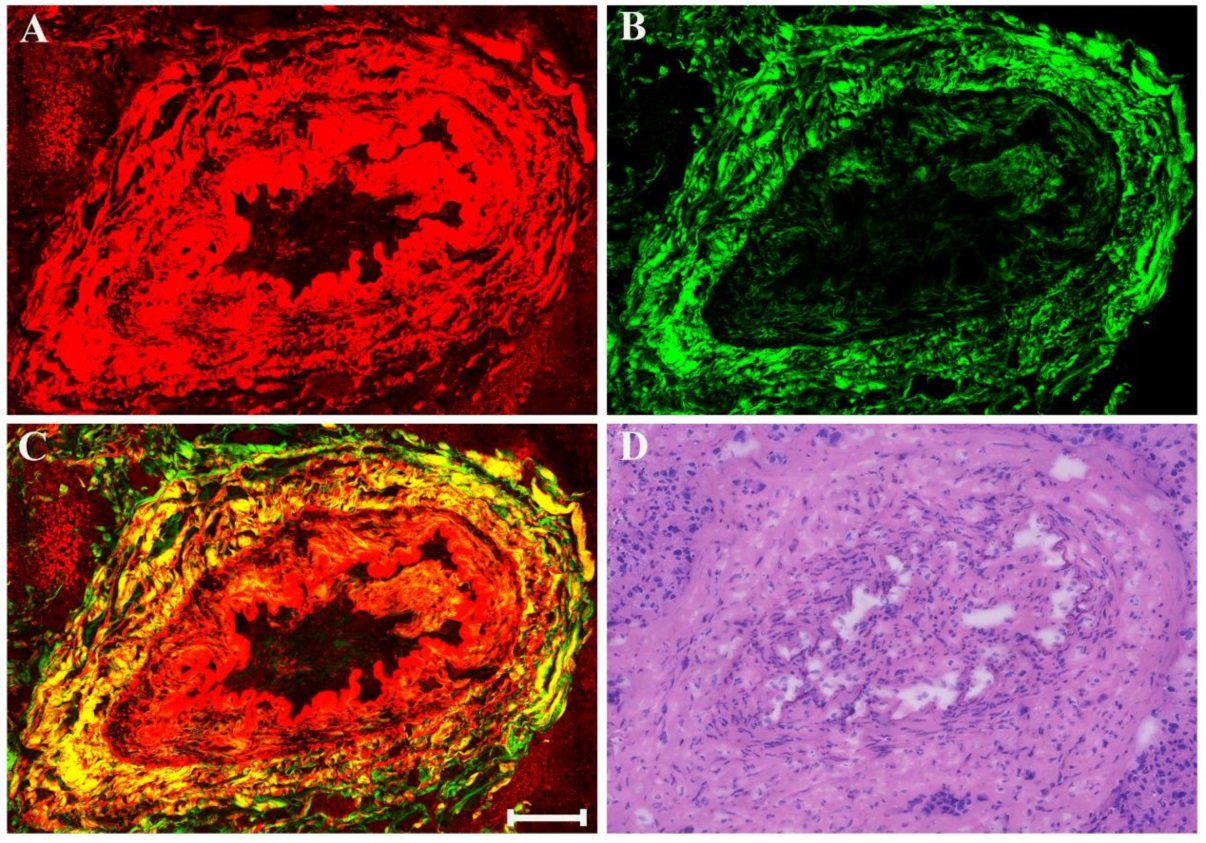
MPM images of vascular hyperplasia in breast cancer after neoadjuvant treatment and corresponding H&E-stained image. (A) TPAF image; (B) SHG image; (C) Overlaid image; (D) H&E-stained image. Scale bar: 100μm.

There may also be myxoid or mucinous variations to the stroma in breast cancer following presurgical chemotherapy, and the representative MPM images of mucinous response are revealed in Figure 6. With the appearance of large amounts of mucus, many tissue components in breast cancer are lost. Only a few broken collagen fibers can be seen by SHG imaging because these mucus cannot generate TPAF or SHG signals. Interestingly, rare residual tumor cells which float in the mucus pool (white arrow in Figure 6C) are detected too. Further, it is also important to be aware of other effects when studying breast carcinoma after presurgical chemotherapy such as vascular hyperplasia, calcification. Drug-induced blood vessel hyperplasia is presented in Figure 7. It can be seen clearly that this vessel grows irregularly and is surrounded by a thick layer of collagen fibers. The lumen almost disappears, which would cause blockage, leading to ischemia and even ischemic necrosis of tissues. Particularly the vascular intima is markedly thickened and malformed that may induce by elastosis.

**Figure 8:**
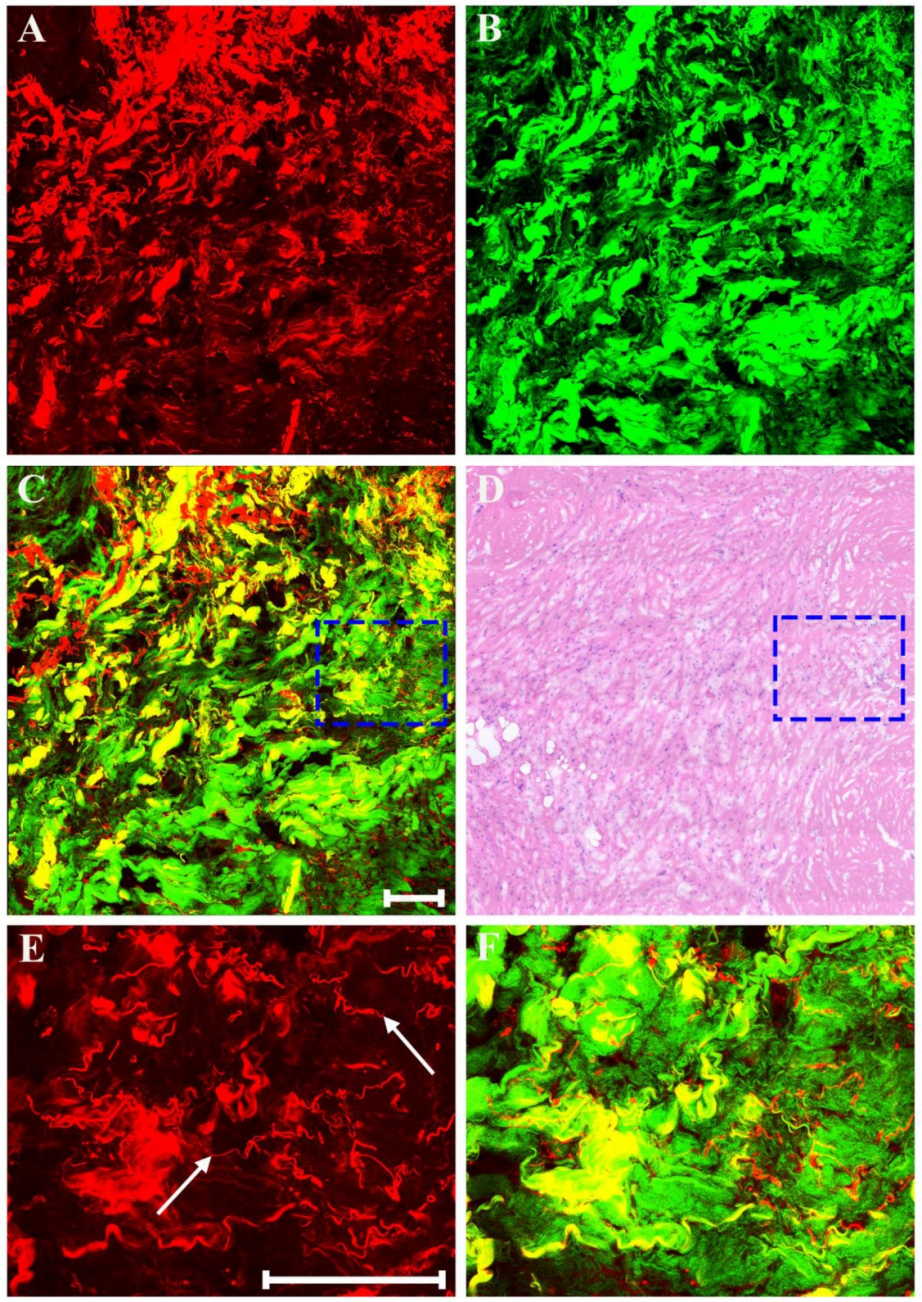
MPM images of severe fibrosis in breast cancer after neoadjuvant treatment and corresponding H&E-stained image. (A) TPAF image; (B) SHG image; (C) Overlaid image; (D) H&E-stained image; and (E-F) Zoom-in TPAF and MPM images of the blue boxed region in (C) respectively. White arrow: elastic fibers. Scale bar: 100μm.

As displayed in Figure 8, neoadjuvant therapy causes complete regression of breast cancer which is characterized by the absence of rare residual tumor cells scattered through the fibrosis. The fibrotic tissues which have replaced the lost tumor tissues are very obvious. That is to say, in this area, there are lots of collagen fibers which are disordered as well as get together, and would be observed more clearly in the magnified MPM image (Figure 8F). A small number of elastic fibers (white arrow in Figure 8E) which can only produce TPAF signal are found too. These fibers look fractured, but are still curly. To summarize, experimental results reveal that MPM can also accurately monitor the various reactions in the stroma after treatment such as fibrotic change, inflammatory cell invasion, myxoid change, vascular hyperplasia, and so on. In this work, all the imaging results acquired from MPM were confirmed by comparing with the digital images of the corresponding H&E-stained sections.

### Quantitative analysis

In general, for most patients, neoadjuvant chemotherapy would reduce the size of primary tumor and the residual tumors often contain large cells with nuclear enlargement, and occasionally the nuclei are angular. Preoperative treatment also often causes a fibrotic response because of the need for tissue repair. However, how to quantify these changes remains a challenge. That is, we are badly in need of some new prognostic biomarkers to verify these patients who have a response to presurgical treatment that may enable clinical doctors to begin to tailor treatment strategy for the individual. Consequently, two endogenous optical biomarkers, that is, the area of cell nucleus and collagen content, are present to quantify changes in cell size and collagen fibers in this study. Automatic image analysis is performed to obtain the results of these two parameters which are shown as a mean value ± standard deviation (SD).

Table 1. displays that the nuclear area from the pre-therapy tumors is 596.56 ± 208.69 (pixel*pixel), while from the post-therapy tumors is 856.22 ± 255.74 (pixel*pixel), and a statistically significant difference (*P*<0.001) is seen between the two sets of data. It is observed that the area of tumor cell nucleus after treatment is significantly larger than that before treatment because the remaining malignant cells would become enlarged with vacuolated cytoplasm. The measurement results also show that the collagen content of breast carcinoma tissues after treatment is significantly higher than that before treatment. More specifically, the collagen content in the post-therapy tumor tissues is 36.10 ± 12.42 (%), while in the pre-therapy tumor tissues is 22.81 ± 10.23 (%). Statistical analysis demonstrates that these two sets of data are also significantly different (*P*<0.001). Our analysis of the data reveals that these optical markers might further help recognize the breast tumor cells that remain after neoadjuvant treatment and monitor changes in the extracellular matrix.

## Discussion

**Figure 9:**
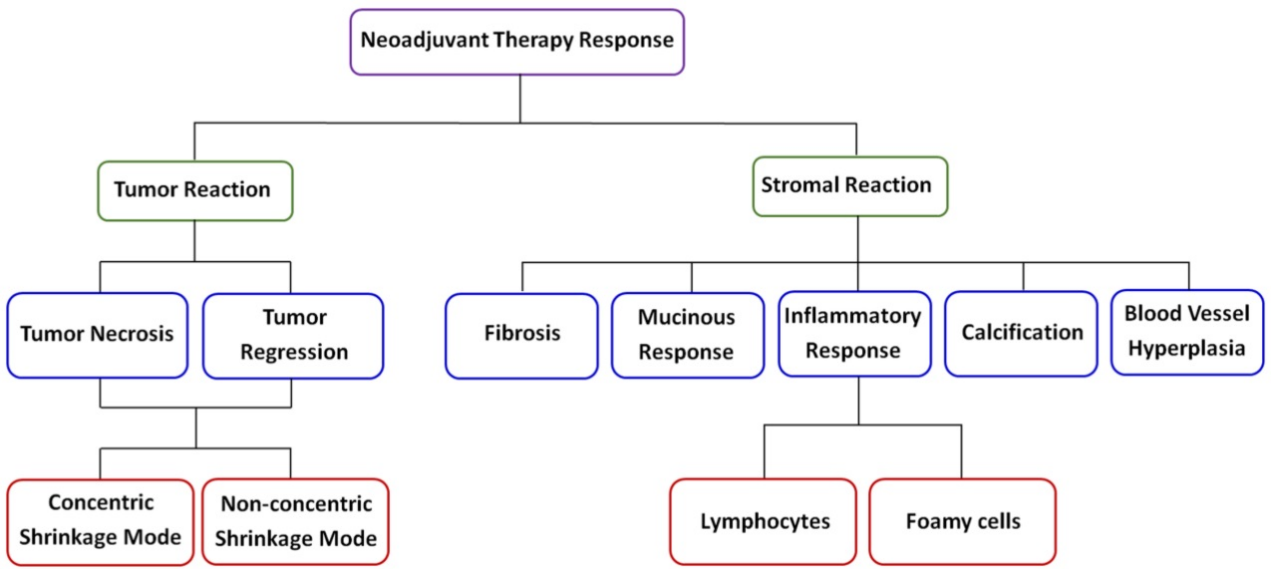
A schematic diagram of neoadjuvant therapy response in breast cancer.

Breast cancer is a systemic disease, and there have been major changes in the management of invasive disease, for instance, neoadjuvant chemotherapy is being increasingly commonly afforded to breast cancer patients, and the trend of this treatment as first-line choice is obvious 23-25. Therefore, when diagnosing such breast tumor samples, it is very important for pathologists to identify the histologic changes induced by the drug. Moreover, for these patients whose tumors show little or no changes after initial therapy, information about treatment response could be used to modify or change therapeutic schedule. Unfortunately, the diagnostic accuracy of preoperative clinical examination remains to be improved at present 26. Clearly, to avoid ineffective treatment or overtreatment, new imaging techniques need to be developed for visualizing therapy response of breast carcinoma following primary cheomotherapy. Therefore, the ability to label-freely image fresh tissue sections at cellular and subcellular levels for evaluation of pathological changes will have great clinical value, and MPM has the full potential to achieve this goal. MPM has the ability to label-freely assess tissue slices without the need of waiting for tissue processing and staining which can help reduce diagnostic time, especially in time-sensitive situations, for example, waiting for the results of intraoperative frozen sections. In this study, we identified distinct patterns of changes in tumor and its microenvironment according to MPM as shown in Figure 9, and all morphologic alterations caused by the drug were confirmed by comparing with the corresponding H&E staining images.

The most important contributors of TPAF signal are mainly from the nicotinamide adenine dinucleotide (NAD) and flavin adenine dinucleotide (FAD) in mitochondria, while extracellular collagen fibers mainly result in SHG signal 27-29. Therefore, TPAF imaging enables researcher to characterize cell viability, morphology, as well as proliferation in both *in vitro* tissues and animal models, while the use of SHG imaging can monitor collagen deposition and extracellular matrix remodeling 30, 31. Our work recognizes several morphologic changes in breast tumor tissues after presurgical chemotherapy, including nuclear change in residual tumor cells, tumor necrosis and regressive changes via TPAF imaging. Tumor regression probably has two patterns: the concentric shrinkage mode and non-concentric shrinkage mode. The non-concentric shrinkage mode was often associated with an increased incidence of tumor multifocality and *in situ* lesions because it may occur by a process of tumor segmentation or fragmentation 8, 12, and even was induced by preoperative treatment in up to 52% of patients 32. Under the circumstances, it is unreliable to rely on clinical examination or mammography to identify suitable candidates for breast-conserving surgery since these techniques are insufficient for detecting these small lesions. Our experimental results also show that residual carcinoma cells are always sparse as well as scattered as single cells with enlarged nuclei in the fibrotic stroma and can be easily detected by TPAF imaging. Additionally, we suggest that if some patients strongly request breast conservation, careful search is required to detect residual carcinoma cells, and the original tumor-bearing area ought to be either removed surgically or subjected to radiotherapy.

Prior studies have shown that alterations in stromal region may be biomarkers of invasion and contribute to understanding the factors that facilitate this process 33-35. Therefore, for the post-treatment breast carcinoma tissues, we also detected changes in the composition and architecture of the extracellular matrix including fibrosis, myxoid change, inflammatory response, and vascular hyperplasia et al. Above all, SHG image can clearly identify the different orientations and distribution of collagen fibers in breast tumor microenvironment, and accordingly, we further suggest that SHG imaging could be used to investigate stromal disorders that are always characterized by abnormal collagen assembly. In a word, we applied TPAF and SHG imaging to evaluate the treatment response of breast carcinoma after preoperative therapy and test results indicated that multimodal multiphoton imaging can discern all the pathological changes, and this paper described our observations on the effect of chemotherapy on a variety of pathological features in a group of breast carcinomas that were clinically responders to treatment. Recently, a portable, intraoperative, real-time, and label-free multimodal multiphoton imaging system was reported as bringing diagnostic potential into operating room as it was successfully used for visualizing the tumor microenvironment shortly in the operating theater once the human breast tissues were surgically excised from patients 36. Hence, multiphoton microscopy might represent a potential tool to be used in monitoring neoadjuvant therapy responses in breast carcinoma which will help doctors determine the most appropriate therapy strategy for each patient, and is possible to promote the development of precision medicine.

### Funding Sources

The project was supported by the National Natural Science Foundation of China (Grant Nos. 81671730, 61972187), the Natural Science Foundation of Fujian Province (Grant Nos. 2019J01269, 2019J01761, 2018J01301, 2018J01183), the Joint Funds for the Innovation of Science and Technology of Fujian Province (Grant No. 2017Y9038), and the Program for Changjiang Scholars and Innovative Research Team in University (Grant No. IRT_15R10).

## Figures and Tables

**Table 1:** Two quantitative variables extracted from MPM images to assess neoadjuvant therapy response in breast carcinoma

Samples	Quantitative parameters	
	Nuclear area (pixel*pixel)	Collagen content (%)
Pre-treatment (n=30)	596.56 ± 208.69	22.81 ± 10.23
Post-treatment (n=30)	856.22 ± 255.74	36.10 ± 12.42
